# The Effects of Qigong on Type 2 Diabetes Mellitus: A Systematic Review and Meta-Analysis

**DOI:** 10.1155/2018/8182938

**Published:** 2018-01-03

**Authors:** Ding Meng, Wang Chunyan, Dong Xiaosheng, Yi Xiangren

**Affiliations:** ^1^Faculty of Physical Education, Shandong Normal University, Jinan 250014, China; ^2^College of Physical Education, Shandong University, Jinan 250011, China

## Abstract

**Objective:**

The purpose of this study was to investigate the effects of Qigong on type 2 diabetes mellitus (DM) using the systematic review and meta-analysis.

**Methods:**

All prospective, randomized, controlled clinical trials published in English or Chinese and involving the use of Qigong by patients with DM were searched in 7 electronic databases from their respective inception to June 2016. The meta-analysis was conducted using the Revman 5.2. The quality of the included trials was assessed using the Jadad rating scale. Two researchers independently completed the inclusion, data extraction, and quality assessment.

**Results:**

Twenty-one trials with 1326 patients met the inclusion criteria and were reviewed. The meta-analysis demonstrated that, compared with no exercise, the Qigong had significant effects on fasting blood glucose (MD = −0.99, 95% CI (−1.23, 0.75), *P* < 0.0001), HbA1c (MD = −0.84, 95% CI (−1.02, −0.65), *P* < 0.0001), and postprandial blood glucose (MD = −1.55, 95% CI (−2.19, −0.91), *P* < 0.00001).

**Conclusion:**

The Qigong training can improve the blood glucose status of the type 2 DM patients and has positive effects on the management of type 2 DM. However, future research with better quality still needs to be conducted to address the effects of Qigong on type 2 DM.

## 1. Background

Diabetes mellitus is a chronic disease caused by inherited and/or acquired deficiency in production of insulin by the pancreas or by the ineffectiveness of the insulin produced. The first WHO Global report on diabetes suggested that the most-to-date number of adults living with diabetes is 422 millions, which has almost quadrupled since 1980. This dramatic rise is largely due to the prevalence of type 2 diabetes and factors driving it include overweight and obesity. The Report on the Status of Nutrition and Chronic Diseases of Chinese residents (2015) indicated that the prevalence of diabetes was 9.7% among adults aged over 18 years, which markedly increased 2.6% in a decade [1].

Modern medical research suggests that improving lifestyle can effectively control and prevent the occurrence of diabetes and its complications. The Diabetes Diagnosis and Treatment Guideline issued by the American Diabetes Association in 2009 stated that exercise prescription is an important treatment on improving lifestyle, and effective exercise can prevent and control the occurrence of type 2 diabetes. Moderate-to-vigorous intensity of aerobic exercise and moderate resistance training can decrease blood glucose and dyslipidemia indicators in patients with type 2 diabetes [2]. Aerobic exercise can improve the correlation between the visceral fat area and HbA1c in middle-aged and elderly obese patients with type 2 diabetes, as well as improving their lipid metabolism, body composition, and cardiopulmonary function, in order to effectively control blood glucose levels [3] and reduce the incidence of middle-aged and elderly obese patients with type 2 diabetes. In summary, exercise has been proven to be an important prescription to help fight against diabetes.

Qigong, a traditional fitness method that originates from ancient China, combines body movement, mediation guidance, respiratory regulation, and so on. The combination of these elements achieves the effect of physical and mental adjustment [4, 5]. Furthermore, Qigong may be capable of helping maintain neuroendocrine balance and blood sugar control. In specific, numerous systematic review studies have been conducted to investigate the effects of Baduanjin on diabetes. Yang et al.'s [6] research included seven clinical trials of Baduanjin on diabetes. The study suggested that the Baduanjin intervention could effectively reduce the fasting blood glucose, HbA1c, and blood lipid levels in patients with diabetes. Yu et al.'s [7] research included ten clinical trials of Baduanjin on diabetes. The results showed that the Baduanjin exercise could regulate blood glucose and blood lipid in diabetes patients. Fengkun [8] concluded that Baduanjin exercise could reduce blood glucose and blood lipid levels in patients with diabetes through a systematic review which included eight trials.

However, there were some inadequate aspects of the previous systematic reviews. First, the existing research included fewer study objects and especially lack of relevant English literatures. Second, existing research merely included Baduanjin Qigong exercise, which did not fully reflect the effects of Qigong on management for diabetes. Therefore, there is a need to include more high-quality researches to further elucidate the role of Qigong in the treatment and management of diabetes.

## 2. Data and Methods

### 2.1. Data Sources and Search Strategies

PubMed, the Cochrane Library, Embase, CNKI, Wan Fang Data, and VIP database were used to search for randomized controlled experiments on Qigong interventions for diabetes. The retrieval duration of these included studies was from the creation of the database to June 2016. At the same time, references in these included studies were also reviewed, with the purpose to avoid omission. The search terms were divided into two major parts: target (diabetes) retrieval and intervention measures (Qigong) retrieval; and the search terms were adjusted according to the specific database. All searches were combined with the theme and free retrieval, and all retrieval strategies were determined after repeatedly performing the preretrieval. These specific retrieval terms include* Qigong*,* Baduanjin*,* Eight-section brocades*,* Wuqinxi*,* diabetes*,* diabetes mellitus*, and* DM*.

### 2.2. Inclusion and Exclusion Criteria

Inclusion criteria included randomized controlled trials, published in Chinese and English language; no restrictions on age or gender of diabetic patients; experimental group taking Tai Chi or Qigong as the major intervention in the exercise of diabetic patients; the control group which does not take any exercise or other aerobic exercises (aerobic exercise except traditional sports including Tai Chi and Qigong: Jogging, walking, etc.) or antiresistance exercises; patients without serious DM-related complications.

*Exclusion Criteria*. Exclusion criteria included experimental groups that used voluntary grouping principles and other nonrandomized controlled experiments; studies that did not include indicators to be discussed in this study; experimental groups that used other main exercises excluding Qigong; patient with DM-related complications.

### 2.3. Trials Inclusion and Data Extraction

Trials inclusion and data extraction process were completed by two researchers (W. C. and D. M.) independently. If there were disagreements between these two researchers during the inclusion and data extraction, a third researcher (Y. X.) would be consulted to analyze and decide whether the trials should be included or the data should be extracted. Data extraction includes information of the first author, publication time, published journals, title of the trials, experimental and control group intervention measures, intervention time, the number of experimental and control groups, and basic information such as the age and gender of patients; diabetes indicators include blood glucose indicators: fasting blood glucose, glycosylated hemoglobin, and two-hour postprandial blood glucose; fasting blood glucose was the first indicator of the study.

### 2.4. Trials Quality Assessment

In the data extraction process, the quality of included studies was evaluated using the Modified Jadad Quality Scale score, which includes random sequence generation and randomization concealment. Each item has a corresponding standard and score: 1–3 score, low-quality research; 4–7 score, high-quality research. Two raters performed the quality assessment independently (M. D. and D. X.). Disagreements were resolved by seeking the opinion of the third rater (Y. X.).

### 2.5. Statistical Analysis

The meta-analysis was performed using Review Manager 5.3 software provided by the Cochrane network [9]. The heterogeneity test* X*2 between the results of the included studies was performed. The heterogeneity results revealed that *P* > 0.1 and* I*2 < 50% and that a fixed effect model should be used. If these heterogeneity test results revealed that *P* < 0.1 and* I*2 < 50%, the source of heterogeneity should be analyzed. The subgroup should be analyzed according to its heterogeneity. If there was no heterogeneity in these subgroups, a fixed effect model should be used. If there was statistical heterogeneity between subgroups, the random effects model for meta-analysis should be used. If data heterogeneity was too large within a subgroup, descriptive analysis should be used. If necessary, a sensitive analysis should be used.

## 3. Results

### 3.1. Trials Search

A total of 202 potentially relevant articles were identified by the database searches. 160 were removed due to duplication, and a total of 21 [10–30] reviews and irrelevant articles were excluded through reading the titles and abstracts. Finally, by screening the full text, 21 articles met the inclusion criteria and were reviewed. One study was published in English and conducted in the US. Another 20 studies were published in Chinese and conducted in China.

### 3.2. Inclusion Research Characteristics and Quality Assessments

A total of 21 randomized controlled trials, with a total of 1,326 subjects, were included. The average age of these subjects ranged within 45.0–68.0 years, with a minimum follow-up time of six weeks and a maximum follow-up time of 12 months. The Modified Jadad score results are as follows: 15 trials were of low-quality, with an average score of 2.7; six were of high-quality, with an average score of 4 (Table 1).

## 4. Outcomes

### 4.1. Group Differences on Fasting Blood Glucose

#### 4.1.1. Qigong Group versus No Exercise Group

**Figure 1:**
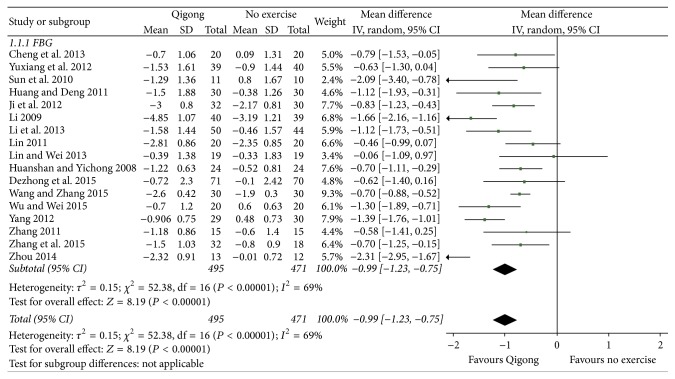
The meta-analysis for comparing fasting blood glucose between the Qigong group and the no exercise group.

Figure 1 shows that data obtained from 17 [10, 12–17, 19, 20, 22, 23, 25, 26, 28–31] related studies (*n* = 966) could be combined. Using the random effect model for data combination, results revealed that the level of fasting blood glucose in the Qigong group was lower than that in the nonexercise group, and the difference was statistically significant (MD = −0.99, 95% CI [−1.23, −0.75], *P* < 0.00001).

#### 4.1.2. Qigong Group versus Other Aerobic Groups

**Figure 2:**
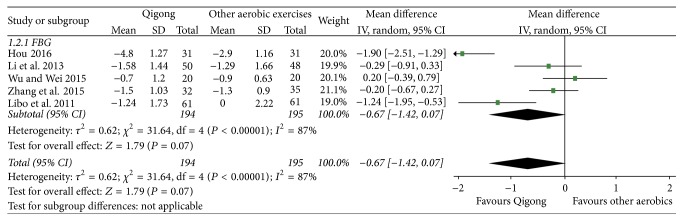
The meta-analysis for comparing the fasting blood glucose between the Qigong group and the other aerobic exercise groups.

Figure 2 shows that the data obtained from five [10, 26–28, 31] related studies (*n* = 389) can be summarized. Using the random effect model for data combination, results revealed that there was no statistically significant difference between the Qigong group and the other aerobic exercise groups (MD = −0.67, 95% CI, [−1.42,0.07], *P* = 0.07).

#### 4.1.3. Qigong Group versus Antiresistance Exercise Group

Only one [29] study (*n* = 22) compared the fasting blood glucose between the Qigong and the antiresistance exercise. The random effects model was used to report the fasting blood glucose, and these results revealed that the fasting blood glucose level in the Qigong group was lower than that in the antiresistance exercise group; and the difference was statistically significant (MD = −1.99, 95% CI [−3.09, −0.89], *P* = 0.0004).

### 4.2. Glycosylated Hemoglobin

#### 4.2.1. Qigong Group versus No Exercise Group

**Figure 3:**
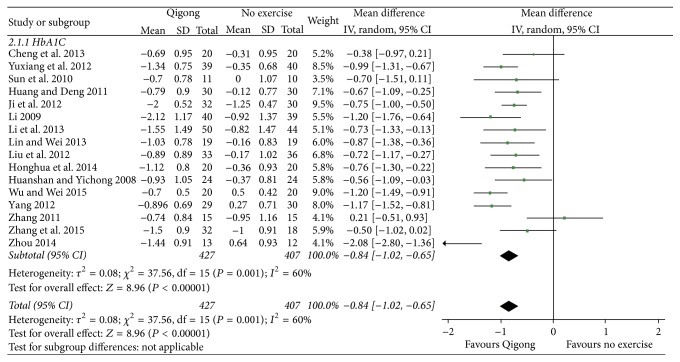
The meta-analysis for comparing the HbA1c between the Qigong group and no exercise group.

Figure 3 shows that the data obtained from 16 [10, 12–22, 28–31] related studies (*n* = 834) could be synthesized. Using the random effect model for data combination, these results revealed that the level of HbA1c was lower than that in the control group, and the difference was statistically significant (MD = −0.84, 95% CI [−1.02, −0.65], *P* < 0.00001).

#### 4.2.2. Qigong Group versus Other Aerobic Exercise Groups

**Figure 4:**
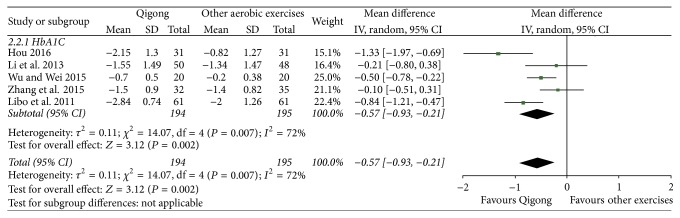
The meta-analysis for comparing the HbA1c between Qigong group and other aerobic exercise groups.

As shown in Figure 4, data obtained from five [10, 26–28, 31] related studies (*n* = 389) could be synthesized. Using the random effect model for data combination, these results revealed that the level of HbA1c of the Qigong was lower than that of the other aerobic exercise group, and the difference was statistically significant (MD = −0.57, 95% CI [−0.93, −0.21], *P* = 0.002).

#### 4.2.3. Qigong Group versus Antiresistance Exercise Group

The random effects model was used to report the HbA1c in one [29] related study (*n* = 22), and the difference was not statistically significant between the Qigong group and the antiresistance exercise group (MD = 0.00, 95% CI [−0.73,0.73], *P* = 1.00).

### 4.3. Two-Hour Postprandial Blood Glucose

#### 4.3.1. Qigong Group versus No Exercise Group

**Figure 5:**
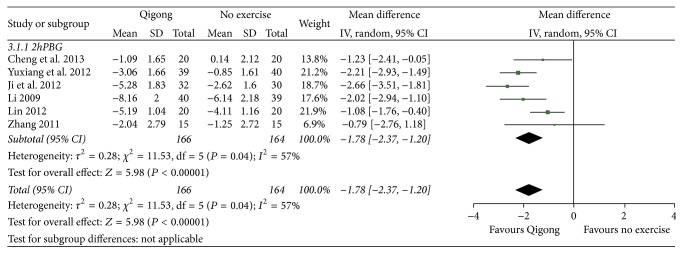
The meta-analysis for comparing the two-hour postprandial blood glucose between the Qigong group and no exercise group.

As shown in Figure 5, the data obtained from six [12, 15, 17, 22, 24, 30] related studies (*n* = 330) could be synthesized. Using the random effect model for data combination, these results revealed that there was a statistically significant difference in the two-hour postprandial blood glucose indicator between the Qigong group and the no exercise group (MD = −1.55, 95% CI [−2.19, −0.91], *P* < 0.00001).

#### 4.3.2. Qigong Group versus Other Aerobic Exercise Groups

Only one [27] study (*n* = 122) reported the comparison of the two-hour postprandial blood glucose between the Qigong and the antiresistance exercise. The random effects model was used to report the two-hour postprandial blood glucose, and these results revealed that the two-hour postprandial blood glucose level in the Qigong group was lower than that in the other aerobic exercise groups; and the difference was statistically significant [MD = −0.84, 95% CI (−1.21, −0.47), *P* < 0.00001].

### 4.4. Publication Bias Evaluation

#### 4.4.1. Publication Bias Evaluation on Comparison between Qigong and No Exercise

The fasting blood glucose for Qigong and nonexercise patients was analyzed through funnel plots, which included 17 trials and 966 objects. These results revealed that the distribution of included studies was asymmetric on both sides of the funnel plots, indicating that it may have publication bias in the comparison of Qigong and nonexercise.

#### 4.4.2. Publication Bias Evaluation on Comparison between Qigong and Aerobic Exercise

The fasting blood glucose was analyzed through funnel plots, which included five studies and 389 subjects. These results revealed that the distribution of the included studies was asymmetric on both sides of the funnel plots, indicating that it may have publication bias in the comparison of Qigong and aerobic exercise.

## 5. Discussion

The purpose of this study was to investigate the effects of Qigong on patients with type 2 DM using the systematic review and meta-analysis. Results suggested that Qigong had a better effect on reducing diabetes patients' fasting blood glucose, HbA1c, postprandial blood glucose, and other indicators, as compared with nonexercise. The results of the present study were consistent with the study conducted by Fengkun [8], Yang et al. [6], Yu et al. [7], and so on indicating that Qigong, as a traditional exercise, can be applied to the management of diabetic patients.

Most traditional oriental exercises such as Qigong, Tai Chi, and yoga have the characteristics of mind-body. According to the recent literature, Qigong, Tai Chi, and yoga played an important role in the management of diabetes. Three systematic reviews [32–34] published reported that yogic practices may promote significant improvements in several indices of importance in type 2 DM management, including glycemic control, lipid levels, and body composition. However, all the reviewers mentioned that methodological quality of the existing clinical trials was low, so high-quality investigations are required to confirm and further elucidate the potential benefits of yoga programs in populations with type 2 DM. The low methodology status of trials also exists in our present study.

Although Qigong is a type of physical and mental exercise that originated in ancient China, which possesses the oriental culture, in the view of modern exercise medicine, it can be concluded that it is a kind of light and moderate intensity aerobic exercise based on the form of Qigong and exercise intensity. light and moderate intensity aerobic exercises can promote cell and tissue metabolism, induce heart blood reflux, improve the body's use of glucose, increase target cell reactivity, improve the body's glucose tolerance, promote HbA1c decomposition, accelerate the combination of hemoglobin and oxygen, and further control blood sugar, thereby reducing fasting blood glucose, HbA1c, and two-hour postprandial blood glucose levels. In the present study, Qigong, as compared with other aerobic exercises, was not found to have any advantages in reducing fasting blood glucose and postprandial blood glucose. However, Qigong is easy to learn, with its easy-to-learn-and-perform nature, Qigong does not require specific site and equipment, both single practice and collective practice are available, and many other modes of aerobic exercises do not possess these features.

In addition, results of this study showed that Qigong demonstrated better control on HbA1c than other aerobic exercise. There are various benefits on increasing HbA1c on human body; it can change the affinity of red blood cells to oxygen, make the tissues and cells hypoxia, and accelerate the formation of cardiovascular and cerebrovascular complications; it may cause glomerular thickening and induce diabetic nephropathy. It can also cause blood lipid and blood viscosity increase. Therefore, lowering the level of HbA1c has a good control effect on the occurrence of severe complications in patients with diabetes. One study [35] showed that walking meditation can reduce HbA1c levels in diabetic patients, while only walking does not. Therefore, we considered that Qigong as an exercise with the combination of movement and meditation could suppress sympathetic activation and thus can improve glycemic control as neutrally mediated vasoconstriction and in the meanwhile reduce glucose delivery and uptake in skeletal muscle [36].

From the Western medicine point of view, the current method of prevention and treatment on diabetes is lifestyle management, including diet and exercise. In terms of exercise intervention for diabetes, Western medicine suggested that patients should be encouraged to engage in moderate intensity aerobic training 3–5 days a week, 30–45 minutes a day, or 150 minutes a week [37, 38]. Furthermore, individualized exercise program should be provided based on the patients' willingness and capability. Quantification and standardization are the main features and advantages of Western medicine; however, mechanical exercise program and monotonous exercise style often make patients feel tired. Qigong, as a traditional exercise belongs to Chinese Tradition Medicine, is not as accurate as Western medicine exercise in terms of the prevention and treatment on diabetes. However, Qigong emphasizes personal understanding and cultural identification of practitioners, while Qigong is an organic combination of movement, mediation, and breathing.

There are limitations to this study that must be considered before the results can be generalized. First, due to the limitation of the searching condition, only the PubMed, the Cochrane Library, Embase, SCI, CNKI, Wan Fang Data, and Wei Pu (VIP) databases were included in this study, and the reviewed studies were bound to be omitted. Second, the included studies generally had major methodology flaws; some researches did not describe the random allocation method and blindness or failed to point out whether there were omitted studies or subjects. The Modified Jadad score in most of the trials was 2, which would affect the credibility of the study results. Third, for included studies that had higher heterogeneity, only the random effect model was available, which may have certain confounding effects on the results. Fourth, it was likely to have publication bias in the included studies. Fifth, only one English published trial was included in our present study, and most included trials were conducted in China; therefore it need more strong evidence to confirm the effects of Qigong on management of type 2 DM in non-Asian population.

## Figures and Tables

**Table 1:** Characteristics and quality assessments of the included trials.

First author, year	Patients (total, E/C)	age (mean (SD), E/C)	Experimental intervention	Control	Duration, sessions with supervision per week, time per session	Modified Jaded score
Pan, 2008	48, 24/24	45 (9)/47 (7)	Qigong	N	24 weeks, NR, 45 min	3
Li, 2009	79, 49/39	57.8 (7.5)/56.5 (6.9)	Qigong	N	6 months, NR, 60 min	2
Sun, 2010	32, 11/10; 11	NR	Qigong	N; PRT	12 weeks, NR, 30 min	3
Huang, 2011	60, 30/30	NR	Qigong	N	6 months, NR, 60 min	2
Lin, 2011	40, 20/20	NR	Qigong	N	2 months, 3, 60 min	3
Zhang, 2011	30, 15/15	NR	Qigong	N	6 months, 2, 60 min	4
Zhou, 2011	122, 61/61	67.4 (9.23)/68.13 (10.64)	Qigong	NR	3 months, 2, 30 min	4
Guan, 2012	79, 30/40	59.20 (8.80)/58.70 (8.30)	Qigong	N	4 months, 1, 60 min	4
Ji, 2012	62, 32/30	60.31 (7.23)/60.26 (7.15)	Qigong	N	2 months, NR, 45 min	3
Liu, 2012	69, 33/36	62.64 (5.98)/65.64 (8.38)	Qigong	N	12 weeks, 1, 40 min	4
Yang, 2012	59, 29/30	NR	Qigong	N	6 months, 2, 60 min	4
Cheng, 2013	40, 20/20	65.55 (6.23)/61.90 (7.07)	Qigong	N	12 months, 3, 60 min	3
Li, 2013	142, 50/44; 48	50.42 (9.68)/52.69 (8.37); 51.62 (7.83)	Qigong	N; jogging or walking	3 months, NR, 30 min	3
Lin, 2013	39, 19/19	64.5 (11.5)/60.8 (12.2)	Qigong	N	6 months, NR, 90 min	2
Liu, 2014	40, 20/20	57 (7)/55 (9)	Qigong	N	6 months, NR, 30 min	2
Zhou, 2014	25, 13/12	NR	Qigong	N	3 months, 1, 30 min	3
Peng, 2015	141, 71/70	NR	Qigong	N	6 months, 1, 30 min	4
Wang, 2015	60, 30/30	61.7 (6.9)/61.3 (8.4)	Qigong	N	3 months, 1, NR	3
Wu, 2015	60, 20/20; 20	63.9 (7.6)/65.3 (6.0); 64.8 (5.8)	Qigong	N; walking	6 weeks, NR, 20 min	2
Zhang, 2015	85, 32/18; 35	57.0 (2.4)/58.2 (1.0); 55.4 (1.7)	Qigong	N; jogging or walking	100 days, NR, 30 min	3
Hou, 2016	62, 31/31	58.82 (6.78)/58.93 (6.47)	Qigong	NR	6 months, 5, 30 min	3

E, experimental group; C, control group; N, no exercise; NR, not reported; min, minute; PRT, progressive resistance training.

## References

[1] WHO | Diabetes http://www.who.int/mediacentre/factsheets/fs312/en/.

[2] Wang L., Gao P., Zhang M. (2017). Prevalence and Ethnic Pattern of Diabetes and Prediabetes in China in 2013. *Journal of the American Medical Association*.

[3] Wang F., Man J. K. M., Lee E.-K. O. (2013). The effects of Qigong on anxiety, depression, and psychological well-being: a systematic review and meta-analysis. *Evidence-Based Complementary and Alternative Medicine*.

[4] Huang C.-L., Tai Y.-K., Yang Y.-H., Wang R.-H. (2012). Efficacy of five-element gymnastics in glucose and lipid control in taiwanese patients with type 2 diabetes. *Research in Nursing & Health*.

[5] Freire M. D. M., Alves C. (2013). Therapeutic Chinese exercises (Qigong) in the treatment of type 2 diabetes mellitus: A systematic review. *Diabetes & Metabolic Syndrome: Clinical Research & Reviews*.

[6] Yang J., Jing-ying L., Wenliang L., Lina M., Hong Z. (2015). Meta-analysis on effects of Baduanjin on patients with type 2 diabetes mellitus. *China Journal of Traditional Chinese Medicine and Pharmacy*.

[7] Yu T.-T., Yu X.-L., Zeng L.-M., Zhou X., Zhao R.-H. (2014). Baduanjin for diabetes: A systematic review. *Chinese Journal of Evidence-Based Medicine*.

[8] Fengkun Z. (2015). *Evidence Based Medicine of Qigong for Its Dominant Diseases*.

[9] Resources for RevMan 5, http://community.cochrane.org/tools/review-production-tools/revman-5/resources-revman-5

[10] Li Z., Zhao Q. L., Zhao L., Liu H. (2013). The advantage of Ba Duan Jin in aerobic therapy for type 2 diabetic patients. *Liaoning Journal of Traditional Chinese Medicine*.

[11] Wang P., Han Q., Li G., Liang R. (2009). Evaluation of varying aerobics interferential effects on type 2 diabetes patients in community. *China Medical Herald*.

[12] Ji X. D., Wang Q. S., Fang C. X. (2012). Effect of exercise therapy on anxiety and depression in the patients with diabetes mellitus. *Practical Geriatrics*.

[13] Tao Z. (2014). Observation of blood sugar and psychological intervention on 25 patients with diabetic depression by Ba Duan Jin of Qigong. *Nei Mongol Journal of Traditional Chinese Medicine*.

[14] Huang R., Deng X. (2011). Treatment of type 2 diabetes with Baduanjin. *Hebei Journal of Traditional Chinese Medicine*.

[15] Cheng W., Wang Z., Tiantian Z. (2013). Effect of the Health Qigong Mawangdui Daoyin on adjuvant therapy for patients with type 2 diabetes. *Modern Journal of Integrated Traditional Chinese and Western Medicine*.

[16] Mingcan Y. (2012). *Observe the Gymnastic Qigong BaDuanJin adjuvant treatment of Type II diabetes*.

[17] Xinghai L. (2009). Effect of Qigong-Baduanjin on Endotheliurn-dependent Arterial Dilation of Type 2 Diabetes. *Journal of Shenyang Sport University*.

[18] Honghua L., Yuan C., Xia Y. (2014). Effect of Baduanjin exercise prescription on physical and mental regulation in type 2 diabetes with anxiety. *Hunan Journal of Traditional Chinese Medicine*.

[19] Huanshan P., Yichong F. (2008). Experimental research of Baduanjin on management of type 2 diabetes mellitus patients. *Journal of Guangzhou University of Traditional Chinese Medicine*.

[20] Lin Y. F., Wei J. (2013). Study of the intervention effect of health Qigong “Ba Duan Jin” on T2DM. *Journal of Longyan University*.

[21] Liu Y., Huo R., Lai Y., Qiuping Y., Caiyun C., Chen Y. (2012). Community-based Study on Effects of Chinese Qigong-Baduanjin on Depression Symptoms and Life Quality of Patients with Type 2 Diabetes Mellitus. *Chinese Journal of Sports Medicine*.

[22] Xin Z. (2011). *Primary Research of Function on Type 2 Diabetic Patients by Gymnastic Qigong Ba Duan Jin*.

[23] Dezhong P., Yue L., Yuquan S. (2015). Influence of eight *R* rigrams boxing on the anxiety state of type2 diabetes mellitus. *Henan Traditional Chinese Medicine*.

[24] Juncheng L. (2011). *Observation of Curative Effect of Chinese Medicine Combined with Ba Duan Jin in the Treatment of Type 2 Diabetes Mellitus*.

[25] Wang C. Y., Zhang H. (2015). Influence of Baduanjin combined with routine treatment on blood glucose level in type 2 diabetic patients. *China Medicine and Pharmacy*.

[26] Jingyan H. (2016). Observation of the nursing effect of Ba Duan Jin exercise on the blood glucose control of patients with type 2 diabetes. *Journal of the Chinese Medical Association*.

[27] Libo Z., Jingqian Z., Xiaoling Z., Zizheng H., Tefeng A., Ming S. (2011). Observation of curative effect of Chinese medicine combined with Ba Duan Jin in the treatment of type 2 diabetes mellitus. *Liaoning Journal of Traditional Chinese Medicine*.

[28] Zhang S., Liu H., Li Z. (2015). Wang Xiumin, Observation of Baduanjin exercise on the improvement of type 2 diabetic peripheral neuropathy. *Hebei Journal of Traditional Chinese Medicine*.

[29] Sun G.-C., Lovejoy J. C., Gillham S., Putiri A., Sasagawa M., Bradley R. (2010). Effects of Qigong on glucose control in type 2 diabetes: A randomized controlled pilot study. *Diabetes Care*.

[30] Yuxiang G., Shanshan W., Mengnan M. (2012). Effect of Baduanjin-based exercise intervention on related parameters in type 2 diabetes patients. *Journal of Nursing Science*.

[31] Wu Y. C., Wei Q. (2015). Clinical observation on the effect of Ba Duan Jin auxiliary therapy on type 2 diabetes mellitus. *Chinese Journal of Gerontology*.

[32] Innes K. E., Selfe T. K. (2016). Yoga for adults with type 2 diabetes: a systematic review of controlled trials. *Journal of Diabetes Research*.

[33] Vizcaino M., Stover E. (2016). The effect of yoga practice on glycemic control and other health parameters in Type 2 diabetes mellitus patients: A systematic review and meta-analysis. *Complementary Therapies in Medicine*.

[34] Thind H., Lantini R., Balletto B. L. (2017). The effects of yoga among adults with type 2 diabetes: A systematic review and meta-analysis. *Preventive Medicine*.

[35] Gainey A., Himathongkam T., Tanaka H., Suksom D. (2016). Effects of Buddhist walking meditation on glycemic control and vascular function in patients with type 2 diabetes. *Complementary Therapies in Medicine*.

[36] Paul-Labrador M., Polk D., Dwyer J. H. (2006). Effects of a randomized controlled trial of transcendental meditation on components of the metabolic syndrome in subjects with coronary heart disease. *JAMA Internal Medicine*.

[37] Type 2 Diabetes in Adults: Management, 2015

[38] Conlin P. R., Colburn J., Aron D., Pries R. M., Tschanz M. P., Pogach L. (2017). Synopsis of the 2017 U.S. Department of Veterans Affairs/U.S. Department of Defense Clinical Practice Guideline: Management of Type 2 Diabetes Mellitus. *Annals of Internal Medicine*.

